# IAVRS—International Affective Virtual Reality System: Psychometric Assessment of 360° Images by Using Psychophysiological Data

**DOI:** 10.3390/s24134204

**Published:** 2024-06-28

**Authors:** Valentina Mancuso, Francesca Borghesi, Alice Chirico, Francesca Bruni, Eleonora Diletta Sarcinella, Elisa Pedroli, Pietro Cipresso

**Affiliations:** 1 Faculty of Psychology, eCampus University, 22060 Novedrate, Italy; v.mancuso95@gmail.com (V.M.); f.bruni@auxologico.it (F.B.); 2 Department of Psychology, University of Turin, 10124 Turin, Italy; francesca.borghesi@unito.it (F.B.); pietro.cipresso@unito.it (P.C.); 3 Department of Psychology, Research Center in Communication Psychology, Catholic Universiry of the Sacred Heart, 20123 Milan, Italy; alice.chirico@unicatt.it (A.C.); eleonora.sarcinella1@unicatt.it (E.D.S.); 4 Department of Geriatrics and Cardiovascular Medicine, IRCCS Istituto Auxologico Italiano, 20149 Milan, Italy

**Keywords:** emotion elicitation, physiological measures, validation, 360° images, virtual reality

## Abstract

Virtual Reality is an effective technique for eliciting emotions. It provides immersive and ecologically valid emotional experiences while maintaining experimental control. Recently, novel VR forms like 360° videos have been used successfully for emotion elicitation. Some preliminary databases of 360° videos for emotion elicitation have been proposed, but they tapped mainly into an emotional dimensional approach and did not include a concurrent physiological assessment of an emotional profile. This study expands on these databases by combining dimensional and discrete approaches to validate a new set of 360° emotion-inducing images. Twenty-six participants viewed 46 immersive images, and their emotional reactions were measured using self-reporting, psychophysiological signals, and eye tracking. The IAVRS database can successfully elicit a wide range of emotional responses, including both positive and negative valence, as well as different levels of arousal. Results reveal an important correspondence between the discrete and dimensional models of emotions. Furthermore, the images that exhibit convergence between the dimensional and discrete emotional models are particularly impactful regarding arousal and valence values. The IAVRS database provides insights into potential relationships between physiological parameters and emotional responses. This preliminary investigation highlights the complexity of emotional elicitation processes and their physiological correlates, suggesting the need for further research to deepen our understanding.

## 1. Introduction

Emotions are complex and individualized experiences that involve several cognitive, behavioral, and physiological processes [1]. They can be defined as temporary states brought on by stimuli and impact a person’s thoughts, feelings, behaviors, and physiological reactions [2]. Various theories have been developed over the years to explain the nature of human emotion. A common distinction at the representational level of emotions is between dimensional models and discrete models. The discrete emotion model considers a select number of fundamental emotion labels [3], describing them as a response pattern that evolved in response to significant environmental events, each with its unique elicitation conditions. Plutchik [4], Izard [5], and Ekman [6], among others, had the greatest impact on the advancement of this field of study. Although many different sets of these fundamental emotions (typically falling into the range of 7 to 14 categories) have been put forth, no agreement has yet been reached on their precise and total number. However, at least five fundamental categories—joy, anger, sadness, fear, and disgust—seem to be shared by most researchers. In terms of automatic emotion recognition, it’s particularly intriguing that these five emotions are universal enough to be cross-cultural. As opposed to the discrete model’s use of emotion labels, dimensional models argue that affective states are best described in terms of a few independent emotional dimensions, typically two or three. Both Mehrabian and Russell (1974) [7] and Osgood, Suci, and Tannenbaum (1957) [8] are frequently cited as having made significant contributions to this field of study. We here refer to these fundamental dimensions as valence (the positiveness or negativeness of an emotion), arousal (a calm-excited scale), and dominance (the perceived degree of control over a social situation). Since emotions play a significant role in human creativity, decision-making, cognition, and brain activity, it is becoming more and more crucial to understand their underpinnings and underlying mechanisms and predict behavioral, experiential, and physiological reactions associated with each emotional episode. Recently, the counterpart of this approach has become the emotion detection field, which relies on findings from emotions inductions in order to reliably detect emotions starting from various stimuli, including videos and physiological signals. In this regard, numerous researchers have tried to find a reliable method to evoke and automatically identify emotional states from objective psychometric measures due to the central role that emotions play in many background processes, including perception, decision-making, creativity, memory, and social interaction. The improvement of a variety of applications, from healthcare to entertainment, and the development of emotion elicitation, recognition, and expression by means of (increasingly reliable and valid) algorithms has resulted in more effective and sensitive technology. To produce ecologically valid affective states and obtain accurate results, effective emotional stimuli are required by relying on core models of emotions (i.e., dimensional or discrete ones).

There are several benefits to combining discrete and dimensional approaches for emotion induction and detection. Discrete models represent emotions in a simple and understandable way, making interpretation and communication simple. They embody the fundamental emotions that are understood by all people. By considering the underlying dimensions of valence and arousal, dimensional models, on the other hand, provide a more subtle and fine-grained representation of emotional experiences. They provide a continuous range of emotions, enabling a more thorough comprehension of the emotional terrain. Researchers can take advantage of each model’s benefits by combining the two approaches [9]. For the accurate categorization of emotions into separate groups, discrete models can serve as a strong basis for emotion detection. On the other hand, dimensional models can support this strategy by capturing the minute differences and complexities within each category. The accuracy and sensitivity of emotion detection algorithms can be improved by this integration, which can result in a more thorough understanding of emotional responses.

In recent years, there has been a notable increase in the use of virtual reality (VR) in psychological research. As a result, VR has been described as a powerful and efficient emotional induction mechanism that can be used to create empathy machines and a range of emotions [10].

VR is a type of immersive technology that uses 3D computer-generated worlds. When a person is fully immersed in VR, they are not actually “there”, but they still feel as though they are. The user’s perception of immersion and a sense of presence in the environment are influenced by different levels of interactivity and immersion [11,12]. Furthermore, virtual stimuli can elicit reflexive reactions that are comparable to those brought on by real-life circumstances [13], which is crucial in explaining its capacity to elicit strong emotions.

Immersion systems come in three varieties: non-immersive, semi-immersive, and immersive. Simpler technology, like a desktop PC, uses non-immersive systems to show environments on a single screen; semi-immersive technologies, like the cave automatic virtual environment (CAVE) or large screens, surround the viewer with colossal projections on the walls or screens; fully immersive devices like Head Mounted Displays (HMDs), for example, are completely immersive systems that separate the user from external world stimuli and offer a full simulated experience, complete with a stereoscopic picture that responds to the user’s head motions.

VR has undergone incredible technological advancements in recent years and is now more affordable and usable, making it more accessible to a larger audience. As a result, it has also been used in experimental psychology, where it has become a useful tool for investigating fundamental cognitive and emotional processes under actual circumstances [14]. First and foremost, the use of VR could increase the ecological validity of psychological science by allowing researchers to study the aforementioned processes under complex, multimodal, and realistic conditions while maintaining strict experimental control.

Moreover, the success of VR can be attributed to its capacity to outperform conventional non-immersive content and to maintain subjects’ immersion in a social setting [15,16,17,18,19], producing immersion [20,21] and a sense of presence [22,23].

The user experiences a sense of presence that makes them feel physically present and gives them the impression that they are interacting and responding in the real world. Some authors contend that presence levels are more closely related to the strength of the emotional state experienced or that various emotional states are connected to various presence levels [24]. In fact, according to studies comparing emotional and neutral environments, the emotional content affected presence, with much lower levels of presence in neutral environments [16,20,22,25].

The novelty of VR for emotion studies stems from its capacity to elicit emotionally immersive content that is ecologically valid in controlled research environments [10,26]. Participants in VR can become fully immersed in the virtual world and experience intense emotional immersion [26]

For technical (simultaneous multimodal recording of bodily activity) and/or ethical reasons (safety), VR environments enable researchers to simulate situations that would be challenging to operationalize in the real world. Examples include driving simulation (for studying anger and aggressive behavior), flight simulation (for studying phobias), height simulation, and crowd simulation (e.g., for stress induction). VR actively engages the entire mind and body to respond to ongoing challenges, in contrast to traditional emotion induction paradigms [26].

VR may be the best option available today for studying emotions and eliciting real-world emotions by effectively evoking emotional reactions with the potential to cause synchronous changes throughout the participant’s entire body.

### 1.1. 360-Degree Media and Emotions Elicitation

VR is a technology that has advanced rapidly in recent years and has a wide range of variations and applications [27]. There are hybrid systems that incorporate VR components that are successful in evoking emotions, though some may disagree on what exactly qualifies as VR. One such system is immersive 360° videos and images featuring a scene in a photorealistic way, which changes according to head movements. Viewers see a 360° view from the point of view where the video was originally recorded. Multiple cameras are used to record a video to create a complete surround scene, which is then digitally stitched together. They can be viewed on flat-screen devices, like a phone or a computer, by dragging the viewpoint with a mouse or a finger. Otherwise, they can be viewed through VR headsets, just like VR games and other interactive experiences. In this sense, producing content for immersive videos is fairly simple, and as a result, there are several types of content fully and freely accessible online [28].

Akin to VR, 360° videos can give users the impression that they are physically present and that they are interacting and reacting as if they were in the real world. This ability to create a sense of presence or the illusion of “being there” in a virtual environment has been usually found to be associated with intense emotional reactions [16,29,30]. The capacity of 360° videos to evoke feelings has made them suitable for both studying emotions more ecologically and also developing datasets of validated stimuli for emotional elicitation.

The environments of VR and 360° videos can also be used to distinguish them: VR is based on computer-generated environments made with specialized software, like Unity software, which enables the creation of any imaginable scenario, from completely fictional to completely real, and the ability to interact with them in a variety of ways. But these environments demand a very high level of technical expertise, including programming abilities. However, there are fewer opportunities for interaction with a 360° video, which is a genuine recording made by a special camera and viewed through a head-mounted display (like a computer-generated video). These videos are, in fact, produced by using 360° cameras to capture live action in the real world, as opposed to VR video games that use computer-generated characters and environments. They differ from 2D videos in that they depict the entire world as opposed to just a portion of it.

The advantages of 360° videos—as opposed to scenes created using computer graphics—are their photorealism and the easiness of creation. In fact, capturing a 360° video simply requires recording a scene with a special camera. These videos’ photorealism and naturalistic tone result in realistic behavior [17], which convey a strong sense of presence [31,32].

Beyond the end-user technologies that enable 360° content creation and consumption, users can publish and disseminate such content on social media platforms like Facebook and video-sharing websites like YouTube. As it has become simpler to produce, share, and consume personalized 360° videos than VR ones, streaming 360° videos has grown in popularity.

In recent years, there have been some attempts to build databases of stimuli for affective computing, particularly emotion elicitation, making use of 360° technologies [33].

Using publicly available datasets to support studies of emotion, mood, and feeling perspectives can be very beneficial for researchers, academics, and clinicians. However, due to a lack of necessary knowledge, the absence of gold-standard equipment, time constraints, a lack of funding, an inadequate controllable environment, the involvement of subjects, and portable devices, creating an empirical database on affective computing is quite difficult. In fact, there are currently few databases produced with VR technologies.

The main characteristics of the existing databases of 360° videos and images are described in Table 1.

Li and colleagues [34] validated online 360° videos available on YouTube measuring affective variables and head movements. Similarly, Jun and colleagues [20] examined a large set of 360° videos, collecting arousal, presence, and head movements, with a larger sample. Marin-Morales and colleagues [35], with the aim of developing a system that can automatically recognize emotion, validated four 360° images, with valence-arousal ratings but also physiological data (EEG and ECG). EEG responses have also been collected with video stimuli instead of images [36].

Overall, few studies examined physiological reactions in immersive virtual environments and, in particular, using a dimensional approach; moreover, only a few sets of emotional stimuli have been validated, and those sets typically contain stimuli with varying degrees of arousal and valence. Only two studies on affective computing have attempted to detect the user’s mood in a virtual environment through physiological signals, and the majority of the stimuli validated are videos, with only four images.

### 1.2. The Current Study

This paper aims to introduce a freely accessible database of 360° images that aims to overcome the limitations of the existing databases. The first innovation is that we measured emotions by integrating dimensional and discrete models. We used Russell’s valence-arousal model, which is frequently used in affect research [37]. This dimensional model posits that affect can be described on a 2D cartesian space divided into four quadrants on the basis of two orthogonal axes: valence and arousal. Each emotional state can be plotted on this 2D plane, relying on valence and arousal associated with it. While valence ranges from unpleasant (such as sadness or stress) to pleasant (such as joy or excitement), arousal can range from inactive (such as uninterested or bored) to active (such as excited). Although these two measures account for the majority of the variation in emotional states, the model can also include a third dimension of dominance. Dominance can range from a sense of helplessness and weakness (lack of control) to a sense of empowerment (in control of everything). We employ the well-known self-assessment manikins (SAM) for self-assessment along these scales [38]. However, a streamlined framework for comprehending emotions is provided by discrete models as well. These models offer a simple and approachable way to conceptualize and discuss emotions by grouping emotions into a small number of distinct categories. We thus used the modified Italian version of the Differential Emotions Scale (mDES; [39]) to gather emotion labels for each 360° image.

Moreover, the analysis of users’ emotional expressions and/or physiological signals is a common method for measuring emotions. To date, most studies on emotion assessment have concentrated on analyzing speech and facial expressions to ascertain a person’s emotional state. Although they have received less attention, physiological signals are also known to contain emotional information that can be used for emotion assessment. We thus included signals coming from the peripheral nervous system (PNS). Overall, we want to create a database of immersive VR images that will be accessible to the public and serve as a potential resource for research on emotion induction, not only in virtual reality. We aim to provide a set of normative emotional stimuli for experimental investigations of emotion and cognition. The goal is to create a large collection of emotionally globally accessible 360° photographs that cover a variety of semantic categories. Second, we are in a unique position to investigate potential relationships between physiological measures and the emotions one experiences while viewing immersive VR because we collected measures of both sympathetic and parasympathetic systems and measures of affect for each stimulus, integrating both discrete and dimensional models of emotions, along with a rate of sense of presence. This dataset joins and enriches existing datasets, providing a different typology of emotional stimuli: the IAPS, the International Affective Digitized Sound system (IADS), and the Affective Lexicon of English Words (ANEW). For researchers, having access to normatively rated collections of emotionally charged stimuli has many advantages. First, it gives researchers more experimental control over the emotional stimuli they use, allowing them to choose ones that are better suited to their particular research questions. This increases the study’s validity and lessens the possibility that unrelated factors will affect the findings. Second, having normative ratings makes it simpler to compare the results of various studies carried out in the same or different laboratories. The integration of findings from various studies can be facilitated by this standardization of stimuli selection, providing a more thorough understanding of the phenomenon being studied. Finally, normative ratings support and facilitate exact replications of studies both within and across research labs. In scientific research, exact replications are necessary to guarantee the accuracy and dependability of the results. Researchers can compare their findings to those of other studies and replicate the same study using the same stimuli in various settings, thanks to the availability of normatively rated stimuli collections. This increases overall confidence in the robustness of the findings. Our participants evaluated forty-six 360° images on valence, arousal, and dominance dimensions using SAM to achieve our objectives. These images can be found online in Zenodo database using this https://doi.org/10.5281/zenodo.7900473 (accessed on 1 May 2024). As participants watched the videos, we also monitored their rotational head movements. This enabled us to correlate the observers’ head movements and affect.

## 2. Materials and Methods

### 2.1. Stimuli

We included images ranging from the lowest to the highest levels of arousal and valence. To prevent cybersickness brought on by scene jumps, we only selected 360° images that were captured with a stationary camera instead of 360° videos. Sources for images include personal contacts and internet searches on websites such as Envato and Flickr. All image creators acknowledged their ownership of the copyright and permitted the videos to be used in current and future research.

Additionally, we conducted an ad hoc selection of high-resolution stimuli, selecting urbanistic (such as squares, streets, and buildings) and naturalistic scenarios (such as views of mountains, lakes, seas, and parks). The images utilized in this study were chosen to encompass urban and realistic (naturalistic) settings. This decision was deliberate for multiple reasons: first, we wanted to guarantee that the content was devoid of any language-related elements, such as semantic and verbal cues, that could potentially impact emotional reactions based on linguistic and cultural differences. Second, to encompass a wide spectrum of emotions, it is important to acknowledge that both naturalistic and urbanistic scenes have the ability to elicit a range of affective states, which can include both positive and negative valence. Third, we aimed to increase the ecological validity of the study, and for this purpose, it is important to utilize realistic settings that participants commonly encounter in their daily lives. This will enable the findings to be more applicable to a wider range of situations. The purpose is to create a range of scenes with varying levels of complexity in order to better study and analyze specific emotional and physiological reactions.

Moreover, there are several justifications for the decision to choose images instead of videos. Film clips from movies, television shows, or music videos, for example, may be processed differently by participants than autobiographical events because they are aware that the events being portrayed are fictional [40]. Participants may not be fully immersed in the experience because of this, which can affect the emotional reactions elicited. The fact that some participants may be familiar with video clips from popular media, like movies and television, may also have an impact on how their memories and emotions are processed [30,41,42]. There may be confounding factors in the research because studies have shown that familiarity with a stimulus can affect how it is remembered and perceived. We were able to produce an experience that is more ecologically valid and closely resembles real-world circumstances by utilizing 360° images. This method enables us to study emotions in a more realistic environment and may result in a deeper comprehension of how emotions function in daily life. For the study, a total of 46 immersive VR clips were chosen. The Supplementary Materials contain a description of each image. All images are available at https://doi.org/10.5281/zenodo.7900474 (accessed on 5 May 2023).

### 2.2. Questionnaires

Before video exposure, participants were required to fill out a questionnaire collecting demographic information, the presence of depression using Beck Depression Inventory (BDI) [43], anxiety using State and Trait Anxiety Inventory (STAI) [44], and the emotion regulation questionnaires (ERQ) [45]. The BDI-II is a widely known 21-item self-report inventory that assesses depression severity over a two-week period. The STAI is a psychological inventory consisting of 40 self-report items on a 4-point Likert scale. Participants fill in the Trait scale composed of 20 items, which includes statements like “I worry too much over something that really doesn’t matter” and “I am content; I am a steady person”.

The ERQ is a 10-item scale assessing respondents’ proneness to regulate their emotions through expressive suppression or cognitive reappraisal strategies. Each question is answered on a 7-point Likert-type scale, with 1 (strongly disagree) and 7 (strongly agree).

After each 360° image, participants were again required to rate on a 5-point Likert scale the valence (“From left to right we progressively move from a negative affective state to a positive affective state. Indicate which image above best represents your mood after having explored the navigable image”), arousal (“From left to right we progressively move from a state of low activation to a state of high activation. Indicate which of the following images best represents your state after having explored the navigable image”), and dominance (“From left to right you progressively move from a situation of no control over your emotions to one with total control. Indicate which of the following images best represents your situation after having explored the navigable image”) using SAM and the sense of presence on a 7-point Likert scale (“I had the feeling of being inside that environment”). Finally, we drew from the modified Italian version of the Differential Emotions Scale (mDES—“Below you will find a series of emotions. Select the one that most reflects your emotional state at this moment”) to outline 11 discrete emotions (enjoyed, angry, awed, contemptuous, disgusted, grateful, guilty, sad, scared, amazed, joyful).

SAM displays a series of graphical representations that vary along the valence, arousal, and dominance axes. These figures have various expressions on a continuous scale. A depressed and unhappy figure appears on one end of the SAM scale for valence, and a happy and smiling figure appears on the other. The SAM scale for arousal shows an excited and interested figure on one end and a calm and relaxed figure on the other. The SAM scale for dominance shows a small figure on one end and a big one on the other.

### 2.3. Physiological Measures

The ongoing experience was assessed through the physiological (skin conductance, blood volume pulse, electromyography, pupil dilation) and behavioral (head movements) measures in Table 2.

We analyzed the Inter-Beat Interval (IBI) derived from the BVP sensor, which corresponds to the interval between RR peaks in the ECG. The IBI, also known as RR, was transformed into an estimation of heart rate (HR) and pulse amplitude (BVP Amplitude), which reflect the proportional rise in blood volume. The continuous BVP record was meticulously inspected to verify the signal quality and the integrity of physiological data on a subject-by-subject. The signal processing involved a 50 Hz notch filter to eliminate power line interference.

The heart rate data of BVP were denoted as HR mean (beats per minute) and RR mean (60,000/HR). For our analysis, we considered only the temporal index, e.g., Mean of Heart rate (mean HR), calculated for each VR environment.

The sampling rate for BVP and SC sensors was set at 128 Hz, and for EMG sensors, the sampling rate was set at 1024 Hz.

In the context of electromyography, we included measurements of frequency components in addition to amplitude in EMG signals. This approach allows us to utilize the extensive information contained within EMG signals, encompassing both their amplitude and frequency characteristics. The investigation of frequency measures, such as Mean Frequency (MNF), was motivated by its recognized usefulness in identifying physiological changes, such as muscle tiredness, and the dynamics associated with emotional expressions [49,50]. MNF analysis provides insights into changes in the frequency spectrum, which might indirectly indicate muscle fiber conduction velocity. This is a valuable indicator of brain activation states during emotional responses. In addition, frequency analysis allows for the investigation of the temporal accuracy of muscle activity, which facilitates a more detailed description of the dynamics involved in emotional expressions [51,52,53]. It is crucial to consider this element, especially when trying to identify distinct emotional states characterized by subtle muscle activations that cannot be easily detected using analyses that solely measure amplitude.

### 2.4. Apparatus

The experiment was implemented using Tobi (TobiiProLab, version 1.145), SteamVR (version 1.17.16), and the Vive Pro Eye, which has Dual-OLED displays with a combined resolution of 2880 × 1600 pixels and 615 PPI providing graphics with super rich colors and contrast.

Two desktop stations (DELL, GS 5590) were set up in the lab room, one for the virtual reality setting and the other one for physiological data.

A Nexus 4 (Bio-trace software, 2008a version) was used to record all physiological measures during the sessions, with a sampling rate of 128 Hz for BVP and SC and 1024 Hz for the two EMG.

### 2.5. Procedure

After signing consent forms and filling out demographic information and the other questionnaires (BDI, STAI, ERQ), participants were required to sit on a chair and complete an initial assessment of their psychological state. This included rating their valence, arousal, and dominance (SAM) on a Likert scale and selecting the emotion that best reflects their current emotional state from a list of 11 emotions provided by the mDES. This information served as a baseline and was conducted by participants answering these questions using their own mobile phones.

Next, sensors for physiological assessment were applied (EMG sensors on the face; BVP and SC on their left hand), and the VR headset was then put on. Participants sat in swivel chairs that allowed them to turn completely around if they desired. While the participants were comfortably seated, the experiments began with a baseline assessment of the psycho-physiological activity, consisting of 3 min with eyes open and 3 min with eyes closed.

The forty-six 360° images were categorized into positive, negative, and neutral groups based on the agreement of five independent raters. The images were then randomized and balanced for valence across 15 different sequences, with each sequence containing 24 images. Each participant watched a different sequence, ensuring an equal number of positive, negative, and neutral images for a total viewing duration of 30 min. Participants were instructed to explore the images freely during the viewing.

**Figure 1:**
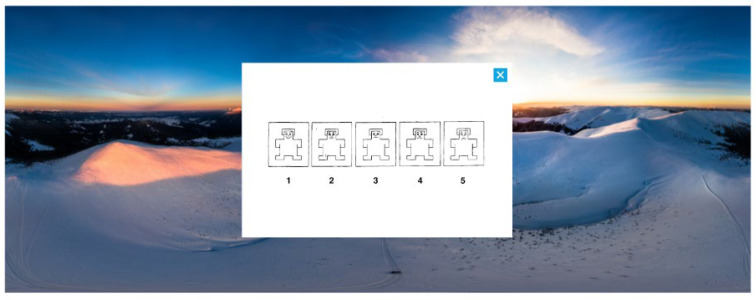
Representation of how the assessment questions (SAM, sense of presence, mDES) appear inside the virtual environments.

After each image was displayed for 30 s, a 5-point rating scale for each SAM dimension was displayed on a white background inside the virtual environment, as shown in Figure 1.

Lower points indicate negative valence/lower arousal/lower control of emotions, and higher points, for positive valence/higher arousal/higher control of emotions.

**Figure 2:**
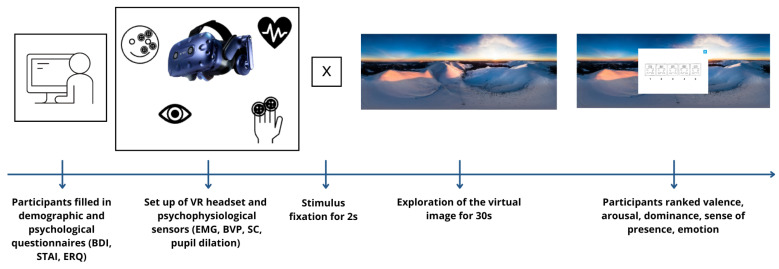
The structure of the experiment: after seeing each image for 30 s, participants had to rate directly in VR their valence, arousal, dominance, and sense of presence on a Likert scale and choose which emotions they felt the most.

Following this, participants rated their sense of presence on a 7-point Likert scale (lower scores indicate less sense of presence). Finally, they had to choose 1 of 11 emotions that best described their emotional state from the list provided within the virtual screen. Figure 2 shows the entire procedure.

### 2.6. Participants

Twenty-six participants, 18 females and 8 males, with a mean of 28.23 years of age (SD = 5.45) and a mean of 16.7 years of education (SD = 2.44), were recruited. All participants voluntarily took part in the experiment without any compensation. All participants reported no neurological or psychological pathologies. All participants gave written informed consent in accordance with the Declaration of Helsinki and received identical instructions.

## 3. Results

### 3.1. Self-Report Measures

At baseline, most participants reported feeling calm (42.3%) and concentrated (19.2%) before stimulus exposure. In fact, they reported a positive tone (mean_valence = 6.08/9, SD = 1.87), a moderate activation (mean_arousal = 4.69, SD = 1.83), and good control of their emotions (mean_dominance = 6.31, SD = 1.81).

Participants reported a mean value of 18.9 (SD = 12.8) for BDI, a mean value of 39.4 (SD = 11.6) for STAI, and a mean value of 33.2 (SD = 4.77) and 14.2 (SD = 6.17) for the cognitive reappraisal and expressive suppression scales of ERQ, respectively.

We found a significant correlation between BDI and arousal (r = −0.580, *p* = 0.002) so that people scoring higher in depression tend to feel more activated during the 360° images view. STAI instead significantly correlated with sense of presence (r = −0.418, *p* = 0.034), meaning that participants experienced less immersion the more anxious they were. Finally, the expressive suppression scale of ERQ correlates with valence (r = 0.446, *p* = 0.022), so people who are more likely to repress their emotions tend to judge images more negatively.

### 3.2. Affective Measures

**Figure 3:**
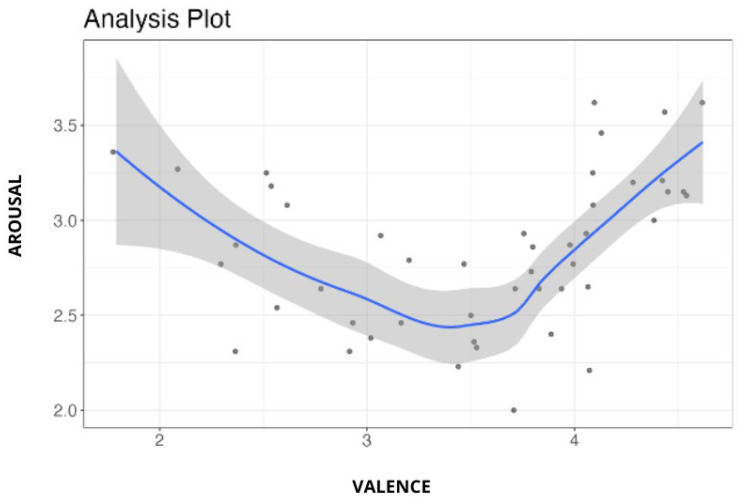
Each image is plotted by mean ratings of arousal (1 = least intense, 5 = more intense) and valence (1 = most negative, 5 = most positive).

The valence and arousal ratings for each image were averaged across participants, and the distribution of these mean ratings can be seen in Figure 3. The mean valence and mean arousal showed a stereotypically asymmetric V-shaped relationship [54,55].

**Figure 4:**
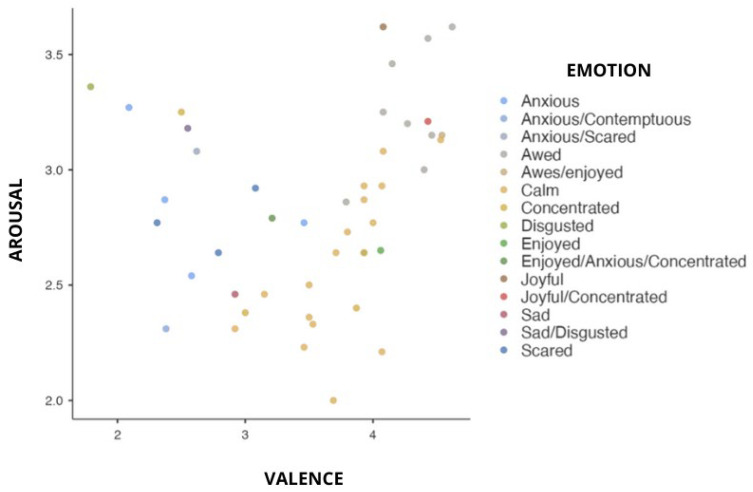
Distribution of 360° images defined by mean arousal and valence ratings.

Figure 4 displays the plots of the immersive 360° images based on the mean ratings of valence and arousal.

Above the midpoint of valence, there is a diverse distribution of video clips with varying both arousal and valence levels. However, only a minority of images caused negative valence. All the images in the database are listed in Supplementary Materials, along with a brief description, valence, arousal, dominance ratings, sense of presence, and physiological measures that correspond.

The immersive images vary on valence ratings (mean = 3.53, SD = 0.751), ranging from 1.79 (negative valence) to 4.62 (positive valence), and on arousal ratings, from 2.00 (low arousal) to 3.62 (high arousal).

By linearly converting a Likert scale from 1 to 5 to a scale from 1 to 9, we were able to compare these stimuli with those of the IAPS.

Similar to IAPS, whose valence ranges from 1.31 to 8.34, IAVRS stimuli covered a broad spectrum (2.58–8.24). IAVRS has fewer stimuli with low arousal than IAPS; in fact, our images range from 3 to 6.24, while IAPS’s range from 1.72 to 7.35.

The results of the one-sample *t*-test showed that the midpoint of the scale was significantly outperformed by the mean score on the Sense of Presence scale (M = 4.86, SD = 0.608) (t(45) = 15.1, *p* < 0.001). As their mean score on the scale was significantly higher than the neutral point of the scale, this suggests that participants felt a strong sense of presence while viewing the immersive images.

**Figure 5:**
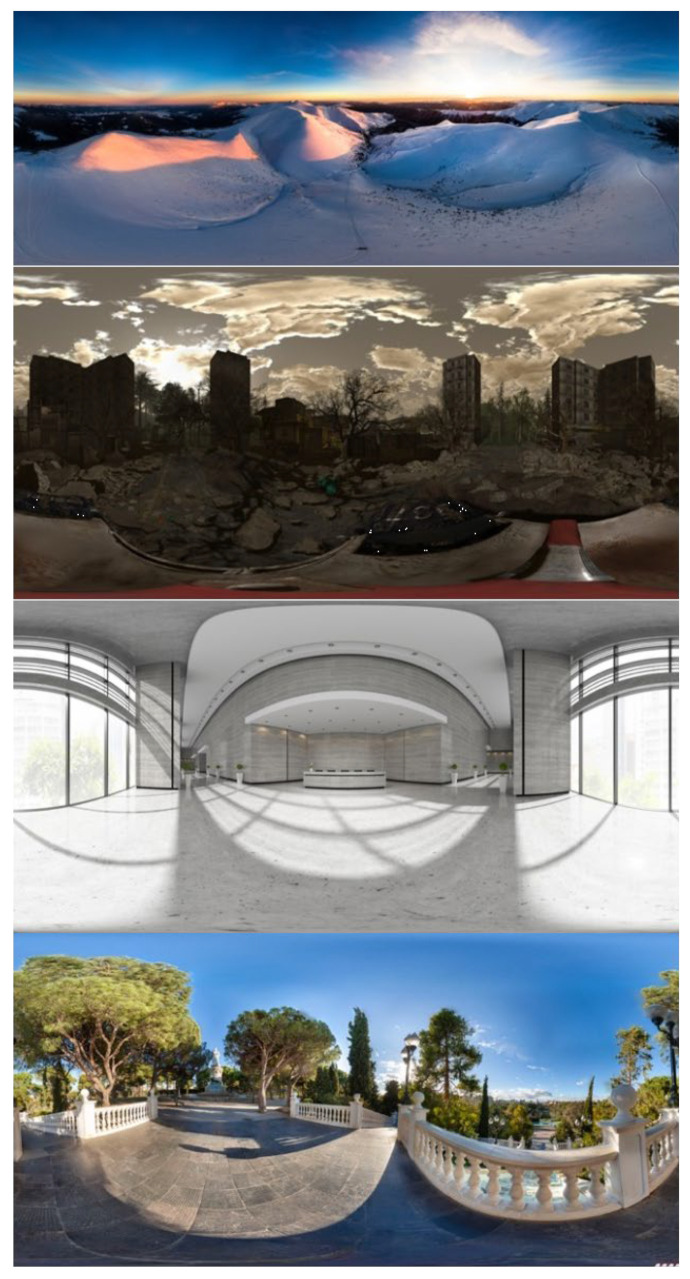
Example of images from IAVRS database that stand out for valence/arousal values. From the top to the botton: positive valence/high arousal image; negative valence/high arousal image; negative valence/low arousal; positive valence/low arousal.

The images where the dimensional and discrete models of emotions converge stand out in terms of arousal and valence values. The word “awed”, which denotes a state of being profoundly impressed or amazed, was specifically applied to the image with the highest positive valence and high arousal (IAVRS 2). This is consistent with the dimensional model’s depiction of an intensely activated and highly positive emotional state. Similar to this, the image with the highest negative valence but high arousal was given the label “anxious”, which encapsulates the sense of unease and distress connected to negative emotions that are characterized by increased activation (IAVRS 29). The image with low valence and low arousal was given the label “concentrated”, implying a focused and attentive state that is consistent with how the dimensional model represents emotions with low valence and arousal (IAVRS 41). Finally, the image that had a positive valence but low arousal was given the label “calm”, which represented a relaxed and calm state (IAVRS 32). These labels serve as an example of how the dimensional and discrete models are compatible with one another because they show how specific emotional terms can be applied to images that represent different valence and arousal combinations. Figure 5 shows these IAVRS images.

### 3.3. Head Movements

We performed a correlation analysis between the images’ head movements data and SAM measures (Table 3).

We also looked at the relationships between valence and arousal and the four measures of head movement (x, y, z, w), including their means and standard deviations for each image.

Our findings showed a significant correlation between arousal and the x, y, and z head movement variables’ standard deviations. More specifically, higher arousal was linked to greater three-dimensional variability in head movements.

### 3.4. Psycho-Physiological Measure

The Zygomatic Major Muscle and Corrugator Supercilii Muscle were specifically mentioned in the results regarding valence. The Supplementary Materials provides for each IAVRS stimulus the mean and standard deviation of skin conductance, Corrugator Supercilii Muscle activity (EMG1_mean_freq and EMG1_amplitude_mean), Zygomatic Major Muscle activity (EMG2_Mean_Freq and EMG2_amplitude_mean),), Heart rate (HR_BVP), right pupil dilation, left pupil dilation, pitch, yaw, roll, and total for head movements (head_x, head_y, head_z, head_w).

Due to technical difficulties, we had to remove SC data from two participants (Participant 1 and Participant 15). During data collection for Participant 1’s EMG, the Biopac device malfunctioned and shut off, whereas Participant 15’s EMG signals were distorted, most likely because of interference.

Additionally, due to signal distortion, most likely brought on by interference, we deleted data from 10 participants for the EMG measures. Overall, excluding these participants allowed us to concentrate on the high-quality data that was left while ensuring the validity and reliability of our data.

Correlation analysis revealed a significant relation between EMG1 mean frequency and amplitude and valence (r = −0.349, *p* = 0.018, *p* = −0.355, *p* = 0.015) so that corrugator Supercilii Muscle activity increased with negative stimuli. This result is in line with earlier studies that suggested EMG is a valid indicator of valence because it captures the level of muscle tension induced by emotional experiences. Moreover, the observed correlation is in line with previous studies suggesting that this muscle is involved in the expression of negative emotions such as disgust, anger, and sadness.

We also observed an interesting positive correlation between Zygomatic Major Muscle activity (mean frequency) and sense of presence during the immersive image viewing (r = 0.316, *p* = 0.032). Increased muscle tension, as detected by EMG, may contribute to a greater sense of immersion and presence in the virtual environment. However, additional study is required to confirm and comprehend this connection between EMG and the sense of presence in immersive virtual environments. Correlations values are shown in Table 4.

Based on the results of the correlation analyses between the variables of valence, sense of presence, arousal, dominance, and the mean and standard deviation of pupil dilation in both eyes, we discovered that valence and sense of presence are positively correlated with each of the four pupil dilation measures, whereas arousal is not correlated with any of them, and dominance is only correlated with the mean pupil dilation in both eyes. This implies that pupil dilation is probably a reliable indicator of emotional intensity that is not solely dependent on arousal. The observed pupil dilation over such a prolonged period may also reflect a more sustained emotional response related to valence rather than a fleeting arousal response, given that participants watched each image for 30 s. On the other hand, arousal is more immediate and is more likely to manifest as quick changes in pupil size. Values of these correlations are shown in Table 5.

SC mean and SC standard deviation did not show any significant correlation with valence (*p* = 0.452, *p* = 0.479), arousal (*p* = 0.695, *p* = 0.710), dominance (*p* = 0.666, *p* = 0.623), and sense of presence (*p* = 0.236, *p* = 0.235), respectively. The same results have been found for BVP: no significant correlation has been found between BVP_HR mean and BVP_HR standard deviation and valence (*p* = 0.787, *p* = 0.489), arousal (*p* = 0.397, *p* = 0.155), dominance (*p* = 0.262, *p* = 0.404), and sense of presence (*p* = 0.106, *p* = 0.178) respectively.

## 4. Discussion

The primary objective of this study was to provide scientifically validated emotional 360° images. The findings of our study offer insightful information about the emotional reactions elicited by immersive stimuli, which has the potential to advance research in the fields of emotional processing and related areas. The emotional experiences of the participants—including their feelings of valence, arousal, dominance, and the emotion they experienced while viewing each image—were used to psychologically validate the images. This study, moreover, significantly advanced the field of emotion induction by integrating both dimensional and discrete models of emotions. Although the dimensional and discrete models are widely acknowledged as complementary frameworks for comprehending emotions in the scientific community, research studies frequently favor one method over the other [34,56,57]. The consistent correlation between high valence images and positive emotional labels like “awe”, “calm”, “joyful”, and “enjoyed”, as well as the correlation between low valence images and negative emotions like “anxious”, “sad”, “disgusted”, and “scared”, demonstrates a clear correspondence between dimensional and discrete emotional models. According to dimensional models, emotions can be represented as a spectrum of valence, from positive to negative. As a result, positive emotional images frequently have high valence ratings, which is consistent with the positive emotional labels suggested by the discrete model. The relationship between the dimensional representation and the negative emotional labels is similar in that negative emotions are linked to low valence. This consistent alignment supports the idea that discrete labels that precisely capture the unique characteristics of emotional experiences along the valence dimension can effectively complement the dimensional representation of emotions.

Data on the participants’ sympathetic and parasympathetic nervous systems were gathered to conduct physiological validation. Due to the immersive nature of these images, we also investigated the participants’ sense of presence as they viewed each one, as this is an important factor in comprehending the participants’ emotional reactions. Our results indicate that the immersive images utilized in this study can elicit a wide range of emotional reactions, including both positive and negative valence and various degrees of arousal. It is important to keep in mind, though, that the distribution of valence ratings tended to lean slightly in favor of the positive. One explanation for the positive valence predominance in our immersive images could be related to the fact that negative emotions are typically harder to elicit than positive ones, and it can be difficult to find images that effectively elicit strong negative emotions while still being morally and emotionally appropriate for participants. Our results are consistent with earlier research [34,58], which reported comparable difficulties in evoking negative emotions.

Interesting findings from physiological data were obtained for our study, particularly about head movements. As expected, we found a significant relationship between arousal and pitch, yaw, and roll standard deviations. Particularly, greater three-dimensional variability in the head movement was linked to higher arousal. This finding suggests that people tend to move their heads more actively and in a wider variety of ways when they are experiencing more intense emotions. These results are consistent with previous literature [58] but in contrast with Li and colleagues’ results [34], which, against their hypothesis, found a significant relationship between the amount of head yaw and valence rating. It is not surprising that our analysis found significant correlations between the standard deviation of head movements and arousal rather than their mean values.

This is so that the dynamic nature of emotional engagement with 360° images can be captured as the standard deviation provides a measure of variability in the data over time. In contrast, the mean values of head movements may not be as instructive as they represent the average amount of movement throughout the entire presentation of the image and do not capture the temporal fluctuations of emotional responses. This is in line with earlier studies that found that physiological response variability, as opposed to average levels, is frequently more strongly related to emotional states.

In fact, Li and colleagues [34] discovered that the average standard deviation of head yaw was a significant predictor of valence. However, we did not find any correlation with valence.

Overall, these findings imply that head movements could be a significant indicator of emotional engagement with 360° images and could have implications for the creation and assessment of immersive virtual reality experiences.

Investigating the relationship between head movements and emotions can reveal important information about how the body reacts to emotional triggers. Researchers’ improved methods for measuring emotional responses and perhaps even the design of more realistic virtual environments may benefit from an understanding of how emotions are expressed through physical movements, such as head movements. Research in this area may also have applications in gaming and VR therapy, where a user’s experience and general well-being can be improved by being able to accurately track and react to their emotional state. Moreover, valence and EMG1 activity in the corrugator supercilia muscle are correlated so that corrugator supercilia muscles became more active in response to unpleasant stimuli. On the other hand, the Zygomatic Major Muscle’s activity had a positive association with the experience of being present while watching immersive images. This implies that increased muscle tension, as seen by EMG, may help a person feel more immersed and present in a virtual environment. These findings support earlier research that showed a connection between emotional experiences and facial expressions [59]. However, it is important to note that SC and HR did not show significant correlations with either valence or arousal.

Moreover, a significant constraint of our study is the lack of respiration measurement, which has the ability to indicate emotional states and initiate alterations in the autonomic nervous system. While acknowledging the importance of including such measurements, our setup did not incorporate direct respiration or electrocardiography (ECG) sensors.

Recent advancements in wearable technology do allow for simultaneous ECG and respiration measurements with minimal interference in user movement and experience. These devices are generally more stable and less prone to noise, even in active or immersive environments like VR, than BVP sensors. Their use might not significantly impede the immersive experience as previously considered. Wearable sensors that are appropriately integrated with VR equipment could potentially be used without diminishing the immersive experience or the integrity of physiological data.

In future research, we should explore the feasibility of incorporating these non-intrusive wearable sensors into VR settings to enhance the collection and analysis of ECG and respiration data. This approach would improve our understanding of the physiological underpinnings of emotional responses in immersive environments, aligning with our commitment to methodological rigor and participant comfort.

The contradiction between experimental control and ecological validity frequently arises in studies of emotion elicitation. Typically, researchers employ straightforward stimuli to focus on a specific process. The integration of multimodal information streams, their dynamic changes over time, and our responses to them are all overlooked by this method. Our collection of immersive images, on the other hand, offers a singular balance of simplicity and immersion. Our database enables a fundamental induction of emotional states that can be examined in conjunction with cognitive processes, in contrast to earlier studies that used semantic or dynamic stimuli like films. Additionally, our choice of naturalistic and realistic settings devoid of verbal and semantic signals aids in the documentation of intra-individual variability in emotions. Even though we did not compare our database to typical 2D images, we contend that immersive images are necessary for emotional induction since they can trigger more powerful emotional reactions [32,59,60]. However, we acknowledge that the absence of a control condition in our study is a limitation. Future research should include a control condition to rigorously compare the effectiveness of 360° images against other emotion-eliciting methods, such as 2D videos, traditional photographs, and other VR content. This would help to account for individual differences and chance, thereby providing a more comprehensive validation of the emotional responses elicited by our database. However, IAVRS also preserves ecological validity by offering a more comprehensive sensory experience, which has been challenging to create with conventional 2D images. In this way, our work significantly contributes to the study of emotion. The validated database of immersive images may have important ramifications for emotion research, but it may also have useful applications in industries like marketing, entertainment, and mental health therapy. These images, for instance, can be utilized to create more immersive and interesting VR experiences that are more likely to elicit powerful emotional reactions from users. Overall, this study offers a new tool for examining the complexity of emotional experiences in immersive virtual environments, making a significant contribution to the field of emotion research.

## Figures and Tables

**Table 1:** Existing databases of 360° videos and images.

Study	Media	Number	Self-Report Measures	Other Measures	Sense of Presence
[34]	videos	73 (15 per person)	Valence, arousal	Head movements	no
[20]	videos	80 (5 per person)	Arousal, simulator sickness, willingness, preference	Head movements	yes
[35]	images	4 (4 per person)	Valence, arousal	EEG, ECG	no
[36]	videos	450 (15 per person)	Motion sickness	EEG	yes

**Table 2:** Physiological and behavioral measures collected.

Signal	Measure	Sensor
Skin conductance (SC)	It depends on sweat gland activity, which is controlled by the sympathetic nervous system. It measures psychophysiological arousal.	Two electrodes were positioned on the palmar surfaces of the left index and ring fingers to record SC. SC, which represents the average of the cleaned signal over a specific experimental epoch, is expressed in microsiemens.
Heart rate (HR) from Blood volume pulse (BVP)	It measures complex cardiovascular activity and collects data on sympathetic and parasympathetic activations.	Placed in the medium left finger, BVP is obtained using a photoplethysmography biosensor, which uses a light-emitting diode to measure variations in blood volume in a particular tissue. The amount of blood saturating particular tissue regions determines how much infrared light is transmitted to the photoplethysmography.
Amplitude and mean frequency of Corrugator Supercilii Muscle	Corrugator activity is not dependent on awareness of the eliciting stimulus [46] and is sensitive to unpleasant stimuli [47].	A surface electromyography (sEMG) biosensor
Amplitude and mean frequency of Zygomatic Major Muscle	Positive and negative emotional valence [47].	A surface electromyography (sEMG) biosensor
Pupil dilation	Psycho-physiological emotional intensity [48].	Vive Pro Eye headset (HTC Corp., Xindian, New Taipei, Taiwan)
Head movements	Pitch is the term for the nodding-like rotation of the head around the *X*-axis. Yaw is the head’s rotation about the *Y*-axis, which refers to turning the head from side to side to say “no”. Roll is the term for the *Z*-axis movement of the head, which is equivalent to tilting the head from one shoulder to the other.	Vive Pro Eye headset

**Table 3:** Correlation analyses between head movements, valence, and arousal.

	Valence	Arousal	Dominance	Sop
Head_x_mean	r = −0.144, *p* = 0.341, 95% CI [−0.416, 0.153]	r = 0.059, *p* = 0.697, 95% CI [−0.235, 0.343]	r = −0.295, *p* = 0.047 *, 95% CI [−0.539, −0.005]	r = −0.012, *p* = 0.938, 95% CI [−0.301, 0.279]
Head_x_sd	r = 0.184, *p* = 0.221, 95% CI [−0.112, 0.450]	r = 0.314, *p* = 0.033 *, 95% CI [0.026, 0.554]	r = −0.159, *p* = 0.292, 95% CI [−0.429, 0.138]	r = −0.108, *p* = 0.188, 95% CI [−0.387, 0.188]
Head_y_mean	r = 0.017, *p* = 0.909, 95% CI [−0.274, 0.306]	r = 0.045. *p* = 0.768, 95% CI [−0.249, 0.331]	r = 0.145, *p* = 0.337, 95% CI [−0.152, 0.417]	r = −0.101, *p* = 0.503, 95% CI [−0.195, 0.380]
Head_y_sd	r = 0.175, *p* = 0.244, 95% CI [−0.121, 0.443]	r = 0.326, *p* = 0.027 *, 95% CI [0.040, 0.563]	r = −0.164, *p* = 0.275, 95% CI [−0.434, 0.132]	r = 0.183, *p* = 0.224, 95% CI [−0.114, 0.449]
Head_z_mean	r = 0.097, *p* = 0.523, 95% CI [−0.199, 0.376]	r = −0.211, *p* = 0.160, 95% CI [−0.472, 0.085]	r = 0.198, *p* = 0.188, 95% CI [−0.098, 0.462]	r = −0.035, *p* = 0.817, 95% CI [−0.322, 0.258]
Head_z_sd	r = 0.110, *p* = 0.466, 95% CI [−0.186, 0.388]	r = 0.469, *p* = 0.001 **, 95% CI [0.207, 0.668]	r = −0.270, *p* = 0.069, 95% CI [−0.520, 0.022]	r = −0.056, *p* = 0.712, 95% CI [−0.238, 0.341]
Head_w_mean	R = 0.113. *p* = 0.455, 95% CI [−0.183, 0.390]	r = −0.033, *p* = 0.830, 95% CI [−0.320, 0.260]	r = 0.131, *p* = 0.387, 95% CI [−0.166, 0.406]	r = −0.086, *p* = 0.569, 95% CI [−0.209, 0.367]
Head_w_sd	r = −0.007, *p* = 0.961, 95% CI [−0.297, 0.284]	r = 0.173, *p* = 0.250, 95% CI [−0.123, 0.441]	r = −0.087, *p* = 0.565, 95% CI [−0.368, 0.208]	r = −0.031, *p* = 0.840, 95% CI [−0.318, 0.262]

Note. * *p* < 0.05, ** *p* < 0.01, *** *p* < 0.001.

**Table 4:** Correlation analyses with EMG.

	Valence	Arousal	Dominance	Sop
EMG1_Mean_Freq_mean	r = −0.349, *p* = 0.018 *, 95% CI [−0.065, −0.580]	r = 0.060, *p* = 0.692, 95% CI [−0.234, 0.344]	r = −0.123, *p* = 0.417, 95% CI [−0.399, 0.174]	r = −0.136, *p* = 0.367, 95% CI [−0.410, 0.160]
EMG1_Mean_Freq_sd	r = 0.158, *p* = 0.294, 95% CI [−0.139, 0.429]	r = −0.077, *p* = 0.611, 95% CI [−0.359, 0.218]	r = 0.187, *p* = 0.214, 95% CI [−0.109, 0.453]	r = 0.118, *p* = 0.435, 95% CI [−0.178, 0.395]
EMG1_Ampl_mean	r = −0.051, *p* = 0.737, 95% CI [−0.336, 0.243]	r = −0.030, *p* = 0.843, 95% CI [−0.318, 0.263]	r = 0.018, *p* = 0.906, 95% CI [−0.274, 0.307]	r = −0.036, *p* = 0.812, 95% CI [−0.323, 0.257]
EMG1_Ampl_sd	r = −0.095, *p* = 0.531, 95% CI [−0.375, 0.201]	r = −0.051, *p* = 0.735, 95% CI [−0.337, 0.243]	r = −0.004, *p* = 0.979, 95% CI [−0.287, 0.294]	r = −0.054, *p* = 0.723, 95% CI [−0.339, 0.240]
EMG2_Mean_Freq_mean	r = 0.248, *p* = 0.096, 95% CI [−0.045, 0.502]	r = 0.072, *p* = 0.633, 95% CI [−0.223, 0.355]	r = 0.185, *p* = 0.218, 95% CI [−0.111, 0.451]	r = 0.316, *p* = 0.032 *, 95% CI [0.555, 0.028]
EMG2_Mean_Freq_sd	r = −0.211, *p* = 0.160, 95% CI [−0.472, 0.085]	r = −0.068, *p* = 0.652, 95% CI [−0.352, 0.226]	r = 0.006, *p* = 0.967, 95% CI [−0.285, 0.296]	r = −0.127, *p* = 0.400, 95% CI [0.170, −0.402]
EMG2_Ampl_mean	r = −0.207, *p* = 0.166, 95% CI [−0.470, 0.088]	r = −0.013, *p* = 0.930, 95% CI [−0.302, 0.278]	r = −0.061, *p* = 0.687, 95% CI [−0.345, 0.233]	r = 0.002, *p* = 0.990, 95% CI [−0.289, 0.292]
EMG2_Ampl_sd	r = −0.355, *p* = 0.015 *, 95% CI [−0.585, −0.072]	r = −0.018, *p* = 0.905, 95% CI [−0.274, 0.307]	r = −0.200, *p* = 0.182, 95% CI [−0.463, 0.096]	r = −0.065, *p* = 0.666, 95% CI [−0.349, 0.229]

Note. * *p* < 0.05, ** *p* < 0.01, *** *p* < 0.001.

**Table 5:** Correlation analysis with pupils’ dilation.

	Valence	Arousal	Dominance	Sop
Pupil_dx_mean	r = −0.669, *p* < 0.001 ***, 95% CI [−0.803, −0.470]	r = −0.128, *p* = 0.397, 95% CI [−0.493, 0.169]	r = −0.319, *p* = 0.031 *, 95% CI [−0.557, −0.031]	r = −0.481, *p* < 0.001 ***, 95% CI [−0.677, −0.222]
Pupil_dx_sd	r = −0.462, *p =* 0.001 **, 95% CI [−0.663, −0.198]	r = −0.058, *p* = 0.704, 95% CI [−0.342, 0.237]	r = −0.280, *p* = 0.060, 95% CI [−0.527, 0.012]	r = −0.364, *p* = 0.013 *, 95% CI [−0.592, −0.083
Pupil_sx_mean	r = −0.674, *p* < 0.001 ***, 95% CI [−0.807, −0.478]	r = −0.084, *p* = 0.580, 95% CI [−0.365, 0.212]	r = −0.337, *p* = 0.060, 95% CI [−0.571, −0.052]	r = −0.514, *p* < 0.001 ***, 95% CI [−0.700, −0.263]
Pupil_sx_sd	r = −0.494, *p* < 0.001 ***, 95% CI [−0.686, −0.238]	r = −0.240, *p* = 0.109, 95% CI [−0.496, 0.054]	r = −0.243, *p* = 0.060, 95% CI [−0.498, 0.051]	r = −0.424, *p* = 0.003 **, 95% CI [−0.636, −0.152]

Note. * *p* < 0.05, ** *p* < 0.01, *** *p* < 0.001.

## Data Availability

The original data presented in the study are openly available in IAVRS database at https://doi.org/10.5281/zenodo.7900474, (accessed on 5 May 2023).

## Supplementary Materials

The following supporting information can be downloaded at: https://www.mdpi.com/article/10.3390/s24134204/s1, Supplementary Materials contain a description of each image. All images are available at https://doi.org/10.5281/zenodo.7900474, (accessed on 5 May 2023). Supplementary Materials shows, for each IAVRS image, mean values and standard deviation for valence, arousal, dominance, sense of presence, emotion, SC, EMG (ampli-tude and mean frequency), HR_BVP, Pupil dilation, Head movements.
